# New Insights to Clathrin and Adaptor Protein 2 for the Design and Development of Therapeutic Strategies

**DOI:** 10.3390/ijms161226181

**Published:** 2015-12-10

**Authors:** Ebbe Toftgaard Poulsen, Agnete Larsen, Alen Zollo, Arne L. Jørgensen, Kristian W. Sanggaard, Jan J. Enghild, Carmela Matrone

**Affiliations:** 1 Department of Molecular Biology and Genetics, Aarhus University, Gustav Wieds Vej, 10C, Aarhus 8000, Denmark; etp@mbg.au.dk (E.T.P.); kws@mbg.au.dk (K.W.S.); jje@mbg.au.dk (J.J.E.); 2 Institute of Biomedicine, Aarhus University, Bartholins Alle’, 6, Aarhus 8000, Denmark; al@biomed.au.dk (A.L.); zollo@biomed.au.dk (A.Z.); alj@biomed.au.dk (A.L.J.)

**Keywords:** Clathrin heavy chain, APP, _682_YENPTY_687_, AICD, AP-2, Alzheimer’s disease

## Abstract

The Amyloid Precursor Protein (APP) has been extensively studied for its role as the precursor of the β-amyloid protein (Aβ) in Alzheimer’s disease (AD). However, our understanding of the normal function of APP is still patchy. Emerging evidence indicates that a dysfunction in APP trafficking and degradation can be responsible for neuronal deficits and progressive degeneration in humans. We recently reported that the Y_682_ mutation in the _682_YENPTY_687_ domain of APP affects its binding to specific adaptor proteins and leads to its anomalous trafficking, to defects in the autophagy machinery and to neuronal degeneration. In order to identify adaptors that influence APP function, we performed pull-down experiments followed by quantitative mass spectrometry (MS) on hippocampal tissue extracts of three month-old mice incubated with either the _682_YENPTY_687_ peptide, its mutated form, _682_GENPTY_687_ or its phosphorylated form, _682_pYENPTY_687_. Our experiments resulted in the identification of two proteins involved in APP internalization and trafficking: Clathrin heavy chain (hc) and its Adaptor Protein 2 (AP-2). Overall our results consolidate and refine the importance of Y_682_ in APP normal functions from an animal model of premature aging and dementia. Additionally, they open the perspective to consider Clathrin hc and AP-2 as potential targets for the design and development of new therapeutic strategies.

## 1. Introduction

While the neuropathological features of Alzheimer’s disease (AD) are well documented, the details of the mechanism have not been clearly defined yet. Although many studies have highlighted the neurotoxicity of Aβ as a possible cause for AD, accumulating evidence points to endocytosis, trafficking and intracellular sorting of Amyloid Precursor Protein (APP) as potential targets to address the pathology in humans [1,2,3,4,5].

We previously reported that replacing Y_682_ (on the _682_YENPTY_687_ domain of APP) by a glycine (Y_682_G) in knock-in mice provokes a premature, age-dependent decline in cognitive, learning, and locomotor performances [6,7]. The Y_682_G mutation stops APP binding to specific adaptors and consequently halts its redistribution inside neurons. These molecular effects result in the phenotype of endo-lysosomal defects and neuronal degeneration in Y_682_G mice [8].

It is our idea that the identification of APP adaptors will provide important insights into the understanding of neuronal functions. Information on regulatory protein interactions might provide novel targets for drug discovery. Indeed, compounds that inhibit or promote specific APP adaptor protein interactions could be targets for AD drug development studies.

In order to identify such adaptors, we performed peptide pull-down (PPD) experiments followed by mass spectrometry (MS) analysis. We found several proteins with a differential ability to bind _682_GENPTY_687_ and _682_YENPTY_687_ peptides, including Clathrin heavy chain (hc), which is known to be involved in the internalization and processing of APP [9,10,11,12]. Additionally, we report that Adaptor Protein 2 (AP-2), one of Clathrin’s adaptor proteins [13,14], was preferentially able to bind Y_682_ when phosphorylated (_682_pY) and such interaction was prevented by Y_682_G mutation. These findings have been substantiated by co-immunoprecipitation analysis from hippocampal tissues of wild type (WT) and Y_682_G mutated mice, confirming the loss of APP binding to both Clathrin hc and AP-2 in Y_682_G mutated mice.

In this article, we provide new insight to the physiopathology of the Y_682_G mice. We depict a scenario in which the loss in the Clathrin hc and AP-2 binding to mutated APP might result in alterations in APP endocytosis and in its consequent accumulation in intracellular compartments. Such alterations lead to a neuronal decline and to the behavioral deficits previously reported in Y_682_G mice [1,6,7,15,16].

## 2. Results and Discussion

### 2.1. Y_682_G Mutation Prevents APP Binding to Clathrin and AP-2

**Figure 1:**
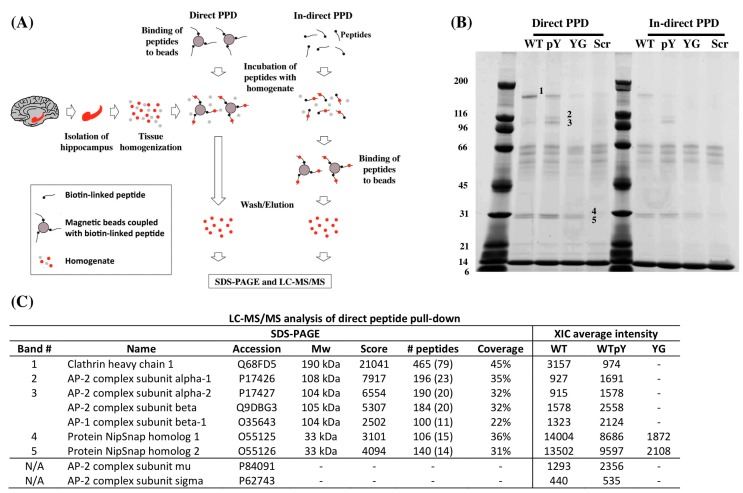
(**A**) Experimental flow-chart of the direct and indirect PPD experiments performed in hippocampal tissues. Hippocampal tissues from three-month-old WT mice was isolated and homogenized for PPD experiments. The direct PPD experiment was conducted by exposing homogenates to beads pre-coupled to peptides. In the indirect PPD experiment, biotin-linked peptides were incubated directly with homogenate before capturing by magnetic beads. After incubation, beads were extensively washed before elution with low pH buffer and SDS buffer. Eluates were either subjected to XIC label-free LC-MS/MS quantification or SDS-PAGE; (**B**) Coomassie blue stained SDS-PAGE of elutes from the direct and in-direct PPD experiments. Numbers indicate proteins bands subjected to LC-MS/MS for identification; (**C**) Table of protein identifications of bands 1 to 5 on the SDS-PAGE, showing the protein name, protein accession number, molecular weight (*M*_W_), mascot software score, number of peptides used for identification and percentage coverage observed by LC-MS/MS. The XIC average intensity displays the relative amount quantified by the XIC label-free analysis. “Scores” refer to the mascot score; “Number of peptides” displays the total number of peptides identified and the number in brackets is the unique number of peptides observed; “Coverage” displays the percentage of the protein sequences that was observed; “XIC average intensity” displays the relative amount bound to each of the mimic peptides in the direct PPD experiment.

In order to identify proteins that differently bind to the _682_YENPTY_687_ domain when Y_682_ is replaced by G_682_ (_682_GENTPY_687_), synthetic peptides mimicking the last 31 amino acid residues of APP were incubated with hippocampal tissues from three month-old WT mice (Table 1). We used either a direct or an indirect pull-down approach (Figure 1a) for our experiments in order to exclude any steric hindrance of APP adaptors to the tagged peptides. Samples eluted from peptide pull down (PPD) experiments were analyzed by extracted ion chromatogram (XIC) label-free quantification using LC-MS/MS and by Coomassie blue stained SDS-PAGE. Interestingly, SDS-PAGE indicated a similar pattern and extent of bands between the direct and in-direct PPD experiments (Figure 1b). Five bands corresponding to proteins that differentially bound the peptides, were subjected to in-gel trypsin digestion and identified by LS-MS/MS (Figure 1c). Band 1 was identified as Clathrin heavy chain 1 (hc). Clathrin hc showed higher preferential binding to the WT peptide than to the pWT peptide and was only lightly detectable in the samples incubated with YG peptide. Bands 2 and 3 were identified as corresponding to AP-2 complex subunits alpha-1, alpha-2 and beta. AP-2 subunits mu and sigma were not detectable from Comassie gel; however, both of them were quantified in the XIC analysis. Notably, band 3 also contains AP-1 subunit beta-1, whereas adaptor protein 3, 4 and 5 (AP-3, AP-4 and AP-5) proteins were not measurable in our experimental conditions. Interestingly, AP-2 complex showed higher preferential binding to the pWT peptide and was not detectable in the samples incubated with YG peptide. Lastly, bands 4 and 5 were identified as protein NipSnap homolog 1 and 2, and they showed equal affinity for both WT and pWT peptides. Additionally, they also interacted with the YG peptides to a lesser extent than seen for the WT peptides. The differential preferences of adaptor proteins for the mimic peptides were supported by the XIC label-free quantification (Figure 1c). A comparison of the average intensities among the different mimic peptides showed that Clathrin hc bound more strongly to the WT peptide than to the pWT peptide. Contrarily, AP-2 showed a higher preference for the pWT peptide than for the WT peptide. Neither Clathrin hc nor AP-2 was found in elutes of the YG or scramble (Scr) peptides. Hence, these results suggest that the Y_682_G mutation affects Clathrin and AP-2 binding to _682_YENPTY_687_.

**Table 1:** Design of synthetic mimic peptides used for pull-down experiments.

Peptide Name	Sequence
**WT**	Biotin-Ttsd linker-AAVTPEERHLSKMQQNGYENPTYKFFEQMQN
**pWT**	Biotin-Ttsd linker-AAVTPEERHLSKMQQNG-pY-ENPTYKFFEQMQN
**YG**	Biotin-Ttsd linker-AAVTPEERHLSKMQQNGGENPTYKFFEQMQN
**Scr**	Biotin-Ttsd linker-LVEYKQREQGFSQPHQTANPFNENAEYTMMK

### 2.2. Clathrin and AP-2 Co-Immunoprecipitate with APP in WT but Not in Y_682_G Mice

**Figure 2:**
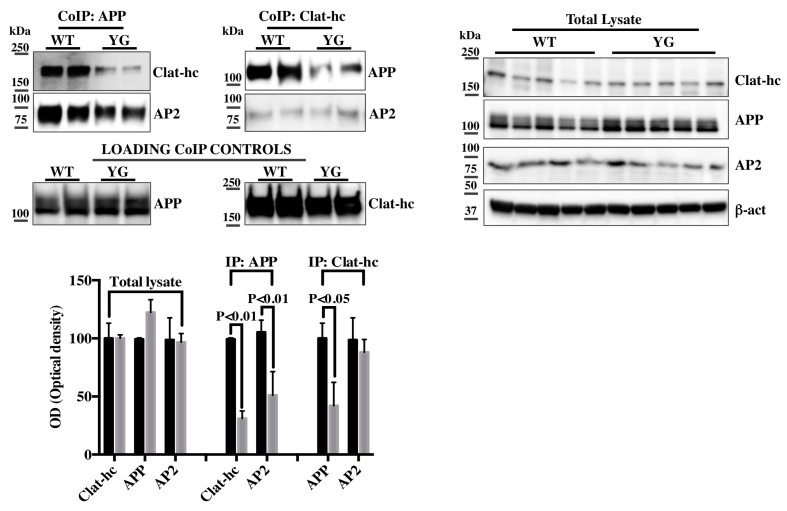
Protein samples from hippocampi of 2 WT or 2 Y_682_G mice were immunoprecipitated with anti-APP (CoIP: APP, **left**) or anti-Clathrin hc (CoIP Clat-hc, **right**) and analyzed with anti Clathrin-hc, or anti-AP-2 and anti-APP and anti-AP-2, respectively. Membranes from *CoIP APP* and *CoIP Clath-hc* were stripped and reprobed with anti APP or anti Clathrin hc antibodies, respectively, in order to demonstrate that equal amount of proteins were immunoprecipitated from both WT and Y_682_G tissues (loading CoIP controls). WB analysis for Clathrin-hc (Clat-hc), APP and AP-2, from total lysates of five different WT and Y_682_G hippocampal tissues (YG) is reported on the right. Densitometric analysis for total lysate and Co-IP is reported below. Data from total lysate samples of WT and Y_682_G mice were normalized to the correspondent β- actin values and expressed as % of WT. Data from IP samples were normalized to the corresponding total protein amount from WT and Y_682_G samples and expressed as % of WT.

Next, we sought to confirm our PPD/MS analyses in hippocampal tissues from WT and Y_682_G mice. For this, we employed three-month-old mice, as we previously reported that synaptic dysfunctions and cholinergic defects commenced at that age in Y_682_G mice [7]. Equal amount of proteins from hippocampal tissues of two WT and Y_682_G mice were immunoprecipitated with either anti-APP (CoIP APP) or anti-Clathrin hc (CoIP Clat-hc) antibodies (Figure 2; *Loading Co-IP controls*) and analyzed with anti-APP, anti-Clathrin hc or anti-AP-2 antibodies. As shown in Figure 2, a reduced APP binding to both Clathrin hc and AP-2 is evident in Y_682_G tissues immunoprecipitated with anti APP when compared to the corresponding WT. Interestingly, samples immunoprecipitated with anti-Clathrin hc showed a reduced Clathrin binding to APP but not to AP-2, indicating that the Y_682_G mutation did not affect the interaction between AP-2 and Clathrin. Notably, both the Clathrin and AP-2 expression levels were not altered from the correspondent hippocampal total lysates (Figure 2). These results suggest that AP-2 likely serves as a hub for recruiting APP from the membrane and forming the clathrin complex. In this respect, it is however worth noticing that further studies will be necessary to clarify the Clathrin-APP-AP-2 interaction *in vivo*.

Notably AP-1 was not identified in samples immunoprecipitated either with anti-APP or anti-Clathrin antibodies, both in WT and Y_682_G tissues, likely suggesting that the amount of AP-1 bound to APP was too small to be detectable by WB (data not shown).

Clathrin requires the presence of several regulatory proteins and adaptors to work properly. In particular, AP-2 and PICALM have been reported to form a molecular complex with the C-terminal residues of APP and to direct APP toward lysosomes for degradation [17]. The expression levels of several Clathrin-regulatory proteins and genes with known functions in Clathrin-mediated endocytosis are altered in AD patients [3,9,10,12]. Indeed, at least three proteins linked to the Clathrin pathway have been associated with sporadic AD: PICALM, BIN1 and CD2AP [18,19,20]. Consistently here, we report that the Y_682_G mutation of APP affects both its binding to AP-2 and Clathrin, suggesting that APP, Clathrin and AP-2 are part of a molecular complex that is essential for correct APP trafficking and avoidance of endo-lysosomal defects and neuronal dysfunction.

## 3. Experimental Section

### 3.1. Tissue Homogenization

Mouse hippocampal tissue from WT mice was homogenized in cold lysis buffer (40 mM, Tris-HCl, 150 mM KCl, 1% Igepal CA630 detergent, pH 7.4) supplemented with complete protease inhibitor cocktail (Roche, Basel, Switzerland), 5 mM EDTA and 1 mM sodium orthovanadate, using a blender. After one hour incubation at 4 °C, under rotation, the homogenate was centrifuged at 20,000× *g* for 10 min to remove cell debris. The protein concentration of the supernatant was estimated using the 2D Quant Kit (GE Healthcare, Little Chalfont, UK). A biotin cleanup was performed on the lysate before pull-down experiments. Lysates were incubated with Pierce Monomeric Avidine Agarose beads (Thermo Fisher Scientific, Waltham, MA, USA) for 1 h to remove endogenous biotin. Beads were subsequently removed by centrifugation.

### 3.2. Biotinylated Peptides

Four synthetic peptides were synthesized by JPT Peptide Technologies GmbH (Berlin, Germany) and used for peptide pull-down experiments. All peptides were synthesized with a N-terminal biotin moiety followed by a Ttsd linker coupled to a 31 amino acid residue-long synthetic peptide (Table 1). The peptides were designed to mimic the last 31 amino acid C-terminal aminoacid residues in APP with (p_682_YENPTY_687_) or without phosphorylation at Y_682_ (_682_YENPTY_687_) (APP residue numbering), and with the Y_682_G amino acid substitution (_682_GENPTY_687_) (APP residue numbering). As a negative control, we used a peptide with a scrambled sequence. Peptides were dissolved in Dimethyl sulfoxide (DMSO) (10 mg/mL) and stored until further use at −20 °C.

### 3.3. Direct Peptide Pull-down

Biotinylated peptides (20 nM) were incubated with 1mg of prewashed Dynabeads M280 Streptavidin (Thermo Fisher Scientific, Waltham, MA, USA) for 2 h at 4 °C under rotation. After five washings in PBS, 0.1% BSA, pH 7.4, dynabeads bound peptides were incubated with 0.5 mg hippocampal lysate for 18 h 4 °C. Beads were then washed five times in PBS, 0.1% Igepal CA630 before elution with 0.1 M glycine, pH 2.8. To increase the yield and to also elute proteins missed by low pH elution, a second elution step was performed consisting in boiling the beads in SDS sample buffer plus 5 mM dithiothreitol.

### 3.4. Indirect Peptide Pull-Down

0.5 mg of lysate were incubated with 20 nM biotinylated peptides for 18 h at 4 °C while rotating. After lysate binding to the peptides, 1mg of prewashed Dynabeads M280 Streptavidin (Thermo Fisher Scientific, Waltham, MA, USA) were added and incubated for additional 2 h at 4 °C. After washing (5 times in PBS, 0.1% Igepal CA630) samples were eluted with 0.1 M glycine, pH 2.8. As an additional elution step, samples were boiled in SDS sample buffer containing 5 mM dithiothreitol.

### 3.5. SDS-PAGE

Samples eluted with SDS sample buffer were loaded on a 5%–15% (*w*/*v*) gradient polyacrylamide gel and electrophoresed using the discontinuous ammediol/glycine buffer system [21]. The gel was stained with Coomassie Brilliant Blue. Bands of interest were cut out and subjected to in-gel digestion using trypsin (sequence grade, Sigma-Aldrich Co, St. Louis, MO, USA). Digested samples were acidified using formic acid and desalted by micro-purification using POROS 50 R2 RP column material (Applied Biosystems, Forster City, CA, USA) packed in gel loader tips. Micro-purified samples were suspended in 0.1% formic acid and kept at −20 °C until LC-MS/MS analysis.

### 3.6. MS Sample Preparation for XIC Quantification

Low pH elutes from peptide pull-down experiments were lyophilized and suspended in 50 µL 8 M urea, 0.2 M Tris-HCl, pH 8.3 plus 5 mM dithiothreitol. Samples were then incubated with 15 mM iodoacetamide for 15 min at room temperature. Reduced and alkylated samples were then diluted five times before incubated with 0.5 µg trypsin (sequence grade, Sigma-Aldrich Co, St. Louis, MO, USA) overnight at 37 °C. Digested samples were micro-purified as described in the SDS-PAGE section.

### 3.7. LC-MS/MS Analysis

LC-MS/MS analyses were performed on an EASY-nLC II system (Thermo Fisher Scientific, Waltham, MA, USA) connected to a TripleTOF 5600+ mass spectrometer (AB SCIEX, Framingham, MA, USA) equipped with a NanoSpray III source (AB SCIEX, Framingham, MA, USA) and operated under Analyst TF 1.6.0 control. Samples were injected and trapped on a C18 pre-column (5 μm, 2 cm × 100 μm I.D., packed in-house). Subsequently, the peptides were eluted to and separated on a 15 cm analytical column (75 μm I.D., packed in-house with RP ReproSil-Pur C18-AQ 3 μm resin (Dr. Maisch GmbH, Ammerbuch-Entringen, Germany) connected in-line to the mass spectrometer. Peptides were eluted at a flow rate of 250 nL/min using a 20 min gradient from 5% to 35% phase B (0.1% formic acid and 100% acetonitrile). An information dependent acquisition (IDA) experiment allowing post-acquisition area-based XIC quantification was used for all LC-MS/MS analyses.

### 3.8. Data Processing

MS files collected for identification of protein bands on SDS-PAGE were converted to Mascot generic format (MGF) using the AB SCIEX MS Data Converter beta 1.3 (AB SCIEX, Framingham, MA, USA), and the “proteinpilot MGF” parameters and searched against the Swiss-Prot Mus musculus database (2015_01) using Mascot 2.5 (Matrix Science, London, UK). Trypsin was employed as enzyme allowing one missed cleavage. Carbamidomethyl was entered as a fixed modification, and oxidation of methionine was entered as a variable modification. The mass tolerances of the precursor and product ions were 10 ppm and 0.2 Da, respectively, and the instrument setting was specified as ESI-QUAD-TOF. The significance threshold (p) was set at 0.01, the ion score expect cut-off at 0.005. Mascot results were parsed using MS Data Miner v. 1.3 [22]. Mascot Distiller 2.5.10 was used for the area-based XIC quantification and the mascot search was performed using the same settings as for protein identification above except that the default average (MD) quantitation protocol was selected, number of peptides used for quantitation was 3, matched rho was 0.8, XIC threshold was 0.3 and isolated precursor threshold was set at 0.7. The area-based XIC quantification was based on the average intensity of the three most abundant peptides per protein.

### 3.9. Immunoprecipitation

For the immunoprecipitation reactions, protein samples were added to Dynabeads-Protein G (30 μg/100 μL) according to the procedure described by the manufacturer (Thermo Fisher Scientific, Waltham, MA, USA) and eluted by heating for 10 min (80 °C) in 0.1 M citrate buffer (pH 2.3). The pH was adjusted by adding 2 M Tris-HCl. Samples were loaded on a 4%–12% SDS-PAGE gel under reducing conditions (Biorad, Copenhagen, Denmark). For IP analysis, we used mouse anti-clathrin (ab24579); rabbit anti-APP (clone Y188) (ab32136); and rabbit anti AP-2 (ab97434) from Abcam (Cambridge, UK).

## 4. Conclusions

Overall, these data underscore the significance of Y_682_ for an appropriate APP binding to specific adaptors and for a correct APP signaling and activity from a knock-in animal model of premature aging and dementia. The loss of APP binding to Clathrin and AP-2 derails APP trafficking and processing and likely explains the premature and progressive neurodegenerative phenotype reported in Y_682_G mice.

Hence, alterations in APP binding to specific adaptors (as a result of genetic polymorphisms in, or near, adaptor proteins, or modifications in adaptor expression or compartmentalization) likely trigger APP missorting in neurons and activate a series of events finally resulting in neuronal degeneration and death.

Overall, our results prospect the possibility that a further analysis of APP adaptors might provide new insights to the design and development of therapeutic strategies.
